# Validation of the behavioral pain scale to assess pain intensity in adult, intubated postcardiac surgery patients: A cohort observational study - POL-BPS

**DOI:** 10.1097/MD.0000000000012443

**Published:** 2018-09-21

**Authors:** Katarzyna Kotfis, Marta Strzelbicka, Małgorzata Zegan-Barańska, Krzysztof Safranow, Mirosław Brykczyński, Maciej Żukowski, Eugene Wesley Ely

**Affiliations:** a Department of Anesthesiology, Intensive Therapy and Acute Intoxications; b Department of Biochemistry and Medical Chemistry; c Department of Cardiac Surgery, Pomeranian Medical University, Szczecin, Poland; d Vanderbilt University School of Medicine, Medicine/Allergy, Pulmonary, and Critical Care, Veteran's Affairs Geriatric Research Education Clinical Center (GRECC) for Tennessee Valley, Nashville, TN.

**Keywords:** behavioral pain scale, cardiac surgery, intensive care unit, pain, sedation

## Abstract

Patients after cardiac surgery experience significant pain, but cannot communicate effectively due to opioid analgesia and sedation. Identification of pain with validated behavioral observation tool in patients with limited abilities to self-report pain improves quality of care and prevents suffering. Aim of this study was to validate Polish version of behavioral pain scale (BPS) in intubated, mechanically ventilated patients sedated with dexmedetomidine and morphine after cardiac surgery.

Prospective observational cohort study included postoperative cardiac surgery patients, both sedated with dexmedetomidine and unsedated, observed at rest, during a nociceptive procedure (position change) and 10 minutes after intervention. Pain control was achieved using morphine infusion and nonopioid coanalgesia. Pain intensity evaluation included self-report by patient using numeric rating scale (NRS) and BPS assessments carried out by 2 blinded observers.

A total of 708 assessments were performed in 59 patients (mean age 68 years), predominantly men (44/59, 75%). Results showed very good interrater correlation between raters (interrater correlation scores >0.87). Self-report NRS scores were obtained from all patients. Correlation between NRS and BPS was relatively strong during nociceptive procedures in all patients for rater A and rater B (Spearman *R* > 0.65, *P* < .001). Both mean NRS and BPS scores were significantly higher during nociceptive procedures as compared to assessments at rest, in both sedated and unsedated patients (*P* < .001).

The results of this observational study show that the Polish translation of BPS can be regarded as a useful and validated tool for pain assessment in adult intubated patients. This instrument can be used in both unsedated and sedated cardiac surgery patients with limited communication abilities.

## 1 Introduction

Epidemiologic studies have shown that critically ill patients after major surgery frequently experience significant pain during their treatment in the hospital.^[1,2]^ The effects of inadequate pain control may be longstanding and severe, and the incidence of chronic pain after cardiac surgery is high, between 21% and 55%.^[3,4]^ Pain following cardiac surgery is a complex syndrome with visceral, musculoskeletal, and neurogenic components.^[3]^ Its treatment must be based upon multimodal analgesia regimens including intravenous opioids, nonopioid analgesics (paracetamol, metamizole), rarely nonsteroidal anti-inflammatory medications (NSAIDS) or anticonvulsants. Effective pain treatment relies on regular and systematic assessment. The gold standard in pain evaluation is patient's self-report using numeric rating scale (NRS) or visual analog scale (VAS). However, pain in critically ill patients often goes unidentified and untreated, because of limited communication abilities in this specific patient population. The most valid and reliable behavioral tools for pain assessment in the critically ill patients who are unable to self-report are critical-care pain observation tool (CPOT) and behavioral pain scale (BPS).^[5]^ Both recommended behavioral scales (BPS and CPOT) have been validated for clinical use in the critically ill adults.^[6]^ This applies to medical, surgical (postoperative), or trauma patients (excluding brain injury) who cannot report pain. Therefore, their validation is still required for other specific patient population, such as cardiac, trauma, or burn patients. One must remember that the use of CPOT and BPS is reliable only in patients with intact motor function and observable behavior.

It must be underlined that other measures of pain assessment, such as changes in vital signs (heart rate, respiratory rate, pupil size, or blood pressure) have been identified as unreliable measures for pain evaluation. Variation in vital signs, especially after a major operation or during critical illness, may be associated with hemodynamic instability or may be the side-effect of concomitant medications. Only systematic pain assessment, including patients’ self-assessment, as well as evaluation by healthcare professionals (physicians, nurses, and physiotherapists) or family members using a validated BPS warrants better identification of patient's needs.^[7]^ Critical care teams must be equipped with dedicated monitoring tools to provide optimal analgesia to all patients.^[5]^ Therefore, the aim of this study was to validate the Polish version of BPS (POL-BPS) in a study including a homogenous group of patients under repeatable intraoperative and postoperative conditions undergoing cardiac surgery.

## 2 Material and methods

### 2.1 BPS translation into Polish

**Figure 1:**
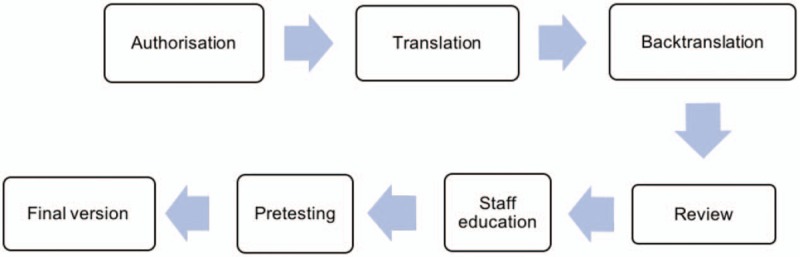
Methodologic steps preceding validation process.

The validation study for POL-BPS is based on previously published recommendations.^[8,9]^ All procedures are briefly summarized in Figure 1. Initially, the process included receiving authorization from the first author of the original scale (J.F. Payen).^[10,11]^ The next steps were: bilingual medical and nonmedical translation, blind backtranslation, review by the expert panel reaching consensus, staff education prior to pretesting, scale pretesting, and preparation of the final version of the test. Afterwards the POL-BPS was published in Polish critical care press and is available for general use with free access.^[10]^ This led to a period of theoretical and practical training regarding the BPS and was the basis for a validation study.

### 2.2 BPS tool construction

Due to the detailed process of translation and back translation, the linguistic and cultural differences were eliminated, and the Polish version of the scale does not differ from the original. The details of the BPS with its 3 domains (facial expression, upper limb movements, and compliance with mechanical ventilation) are depicted in Table 1. The rater can appoint between 1 and 4 points in each category to a total of between 3 and 12 points.^[11]^

**Table 1:**
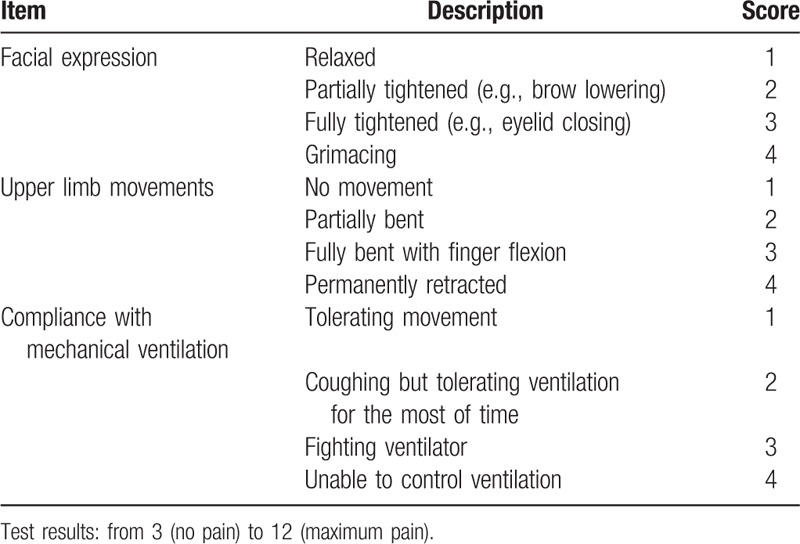
Behavioral pain scale.

### 2.3 Ethical concerns

The Bioethical Committee of the Pomeranian Medical University approved the study and waived a need to obtain consent from participants of this study due to its observational (noninterventional) character, waiver number: KB-0012/180/05/17. Both patients’ self-report of pain as well as behavioral scale pain assessments formed part of the routine care performed by the bed-side nurses in the postoperative intensive therapy unit. The main investigator re-evaluated inclusion and exclusion criteria for all patients enrolled into the study after initial evaluation by 2 members of the study team.

This prospective observational cohort study underwent registration at the ClinicalTrials.gov website and received acceptance (identifier: NCT03127306).

### 2.4 Study population

Patients who fulfilled inclusion criteria were enrolled into this prospective observational cohort study on the 6-bed postcardiac surgery unit and a 6-bed cardiac intensive care unit (ICU) at a tertiary teaching hospital in June and July 2017. All patients underwent a planned cardiac operation with the use of cardiopulmonary bypass (CPB) according to standard practice. Anesthesia was performed using general anesthetic technique with intravenous induction using fentanyl, etomidate, and pancuronium and maintenance of anesthesia using sevoflurane and fentanyl, as per local protocol. All patients were transferred to the postoperative unit where analgesia and sedation was initiated. The unit has implemented a minimal analgesia protocol for intubated postoperative patients, including morphine intravenous infusion with the concomitant use of nonopioid analgesics (metamizole or paracetamol).

### 2.5 Inclusion criteria

**The inclusion criteria were as follows:**
• Age 18 years and above
• Ability to understand Polish language
• Intubated with mechanical ventilation (controlled modes, spontaneous modes)
• Richmond agitation sedation scale (RASS) no lower than −2
• Unrestricted sight and hearing
• Planned cardiac surgery
• No body position change restrictions

### 2.6 Exclusion criteria

- Requirement for deep sedation: severe respiratory failure with patient-ventilator dyssynchrony, preventing awareness during neuromuscular blockade, patient with status epilepticus, surgical conditions requiring immobility, and intracranial hypertension
- Facial trauma or abnormality (unable to evaluate facial expression)
- RASS −4 or −5
- Neurologic or psychiatric disorders
- Regular narcotic users
- Chronic pain syndromes
- Emergency operation

### 2.7 Study measures

During the study, pain assessment consisted of NRS and BPS for each patient, collected at prespecified time-points. Each NRS result was obtained from the patient by the bedside nurse. The BPS was performed by 2 ICU Registered Nurses (rater A and rater B) trained in the use of BPS and using it on regular basis in the ICU. Both raters (rater A and rater B) were blinded to each other and to the NRS result collected from the patient by the bedside nurse. They were supervised by the primary investigator who also verified inclusion criteria. All pain assessments were carried out during rest and routine nursing procedures in the postoperative cardiac unit and cardiac ICU with the nociceptive procedures (NPs), including patient positioning and turning in bed.

Two sets of assessments were performed during prespecified timepoints: at rest 5 minutes prior to the NP, during the NP, and 15 minutes after the NP. Each evaluation consisted of 2 elements: self-report of pain by the patient (using NRS) and BPS assessment carried out by 2 blinded observers. The study procedures were performed in 2 clinical situations: set I in patients sedated with dexmedetomidine (0.4 μg/kg/h) at 3 hours after the procedure and set II in patients unsedated with dexmedetomidine at 6 hours after the procedure. Analgesia was provided as per local protocol with a continuous intravenous infusion of morphine (0.05–0.1 mg/kg/h) and coanalgesia with either paracetamol (1 g every 6 hours) or metamizole (1 g every 6 hours) for opioid sparing effect. Therefore, a total of 6 assessments were performed by each rater (details are depicted in Table 2). The assessments were as follows: series I was performed in patients sedated with dexmedetomidine: T1 = 5 minutes before NP (pre-NP1); T2 = during NP (NP1); T3 = 10 minutes after NP (post-NP1); and series II was performed in unsedated patients: T4 = 5 minutes before NP (pre-NP2), T5 = during NP (NP2), T6 = 10 minutes after NP (post-NP2).

**Table 2:**
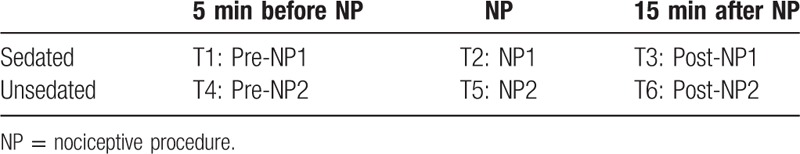
Timepoints for behavioral pain scale and numeric rating scale assessment.

We collected basic demographic data including: age, sex, type of operation, type of mechanical ventilation, use of opioid-sparing coanalgesics during the study. Both arousal and sedation depth were performed prior to each set of BPS assessments using the Polish version of RASS, scoring the patients between −5 (no reaction to voice or touch) to +4 (combative patient). The target RASS was 0 (calm, cooperative patient) and only patients with points more than −3 were included in the study. ICU delirium screening was done using the Polish version of confusion assessment method for ICU (CAM-ICU). Each patient was asked to self-report pain using the NRS using a large-scale print-out and indicate a number between 0 (no pain) and 10 (the worst pain ever).

### 2.8 Statistical analysis

Descriptive statistics were used to analyze the baseline characteristics of the study group and were shown as either mean ± standard deviation (quantitative parameters) or numbers and percentages (qualitative parameters). Standardized Cronbach alpha was used as an internal consistency estimate of reliability for BPS domains. Interrater reliability for BPS was calculated using intraclass correlation coefficient (interrater correlation [ICC] 3,1). Referring to published studies, the sample size required for validating BPS assuming precision of ICC 3,1 at 0.85 ± 0.10 was established between 55 and 65 patients.^[5]^ Spearman rank correlation coefficient (Rs) was used for testing criterion validity between BPS and NRS scales and to analyze correlations between NRS and BPS results and physiologic parameters. Statistical significance was established at *P* < .05. To address the issue of multiple testing, we calculated Bonferroni-corrected threshold *P*-value. Since overall 52 tests were performed to calculate *P*-values presented in Tables 3 and 4, the associations with *P* < .05/52 = 0.00096 were considered to be significant after the correction for multiple testing. The statistical analysis was performed with the use of Excel (Microsoft) and Statistica 13 with Medical Bundle 4.0 (StatSoft Inc., Tulsa, OK).

**Table 3:**
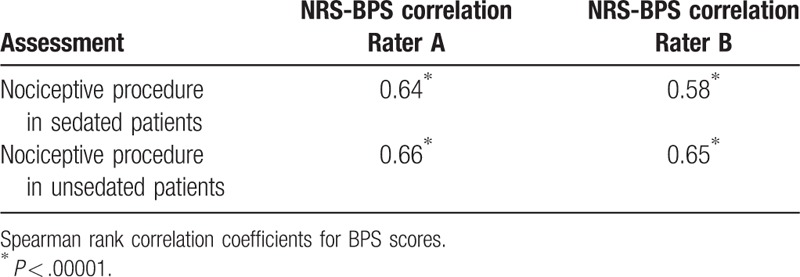
Correlation between behavioral pain scale (BPS) and self-reported pain (NRS) for rater A and rater B, n = 59.

**Table 4:**
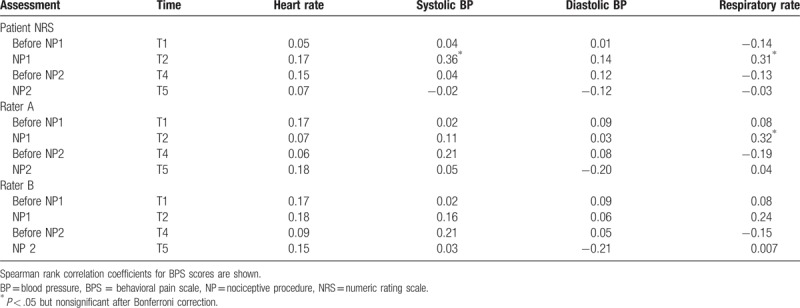
Correlation between physiologic parameters and pain scores (patient NRS) and BPS for rater A and rater B.

## 3 Results

During the 2-month study period 186 patients underwent cardiac surgery with the use of cardiopulmonary by-pass and were screened for eligibility. Out of this group only 80 patients met inclusion criteria and further 21 were excluded from participation in the study (7 = required deep sedation due to intraoperative complications, 6 = underwent an emergency procedure, 4 = chronic pain syndromes, 3 = psychiatric disorders, 1 = required deep sedation secondary to status epilepticus). Altogether the data were completed for 59 patients who were included into the study, with a mean age of 67 years ± 9.6, predominantly males 75% (44/59). All patients underwent an open heart surgery with the use of CPB. Majority of the patients underwent coronary artery bypass grafting (CABG), 46% (27/59), followed by valve replacement 36% (21/59) and combined valvular intervention + CABG (19%, 11/59). All patients were intubated and mechanically ventilated in the postoperative period, mostly using synchronized intermittent mechanical ventilation mode (SIMV), 93% (55/59). We also found that ICU delirium was present in nearly 41% (24/59) of patients, who screened CAM-ICU positive. All patients received intravenous morphine and dexmedetomidine infusion during T1 to T3 and only morphine infusion during T4 to T6. For opioid-sparing effect coanalgesics were administered with frequencies as follows: paracetamol 29% (17/59) and metamizole 71% (42/59). All the baseline data for our study population are shown in Table 5.

**Table 5:**
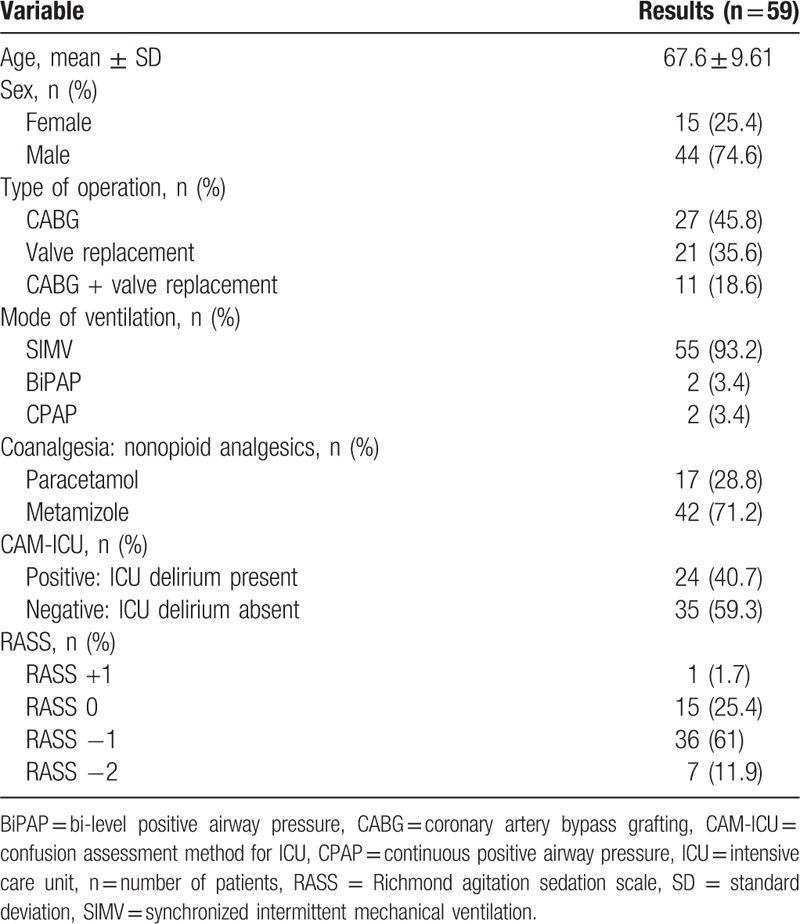
Baseline characteristics of the Polish version of behavioral pain scale study group.

### 3.1 Internal consistency for POL-BPS

Standardized Cronbach alpha values for T2 and T5 assessment times (NP) are presented in Table 6, separately for rater A and rater B. Similar values were not calculated for assessment before or after NP since at least 1 BPS domain showed zero variance (the lowest possible score value: 1 point for all patients).

**Table 6:**
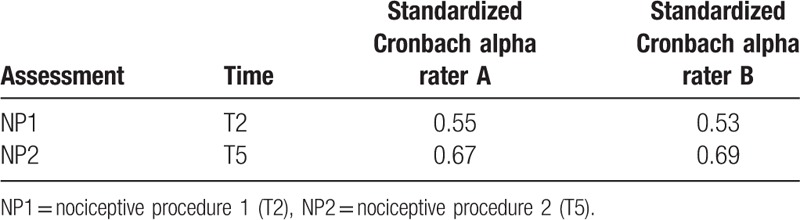
Standardized Cronbach alpha values for rater A and rater B, reflecting internal consistency of behavioral pain scale domains assessed during NPs.

### 3.2 Interrater reliability for POL-BPS

Altogether 708 assessments were performed to calculate the interrater reliability (59 patients × 2 raters × 6 assessment times). The intraclass correlation coefficients (ICCs) were above >0.86 during all assessment times for both raters (rater A and rater B). Therefore, high intraclass correlation coefficients were measures of interrater reliability of the POL-BPS in both sedated and unsedated postcardiac surgery patients. These data are shown in Table 7.

**Table 7:**
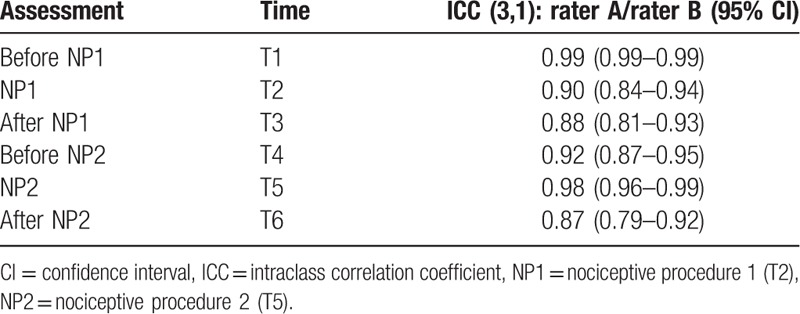
ICC and their confidence intervals for rater A vs rater B at each assessed timepoint.

### 3.3 Discriminant validity for POL-BPS

Concomitant assessments of patients’ self-reported NRS and BPS evaluation performed by rater A and rater B are shown in Table 8. The pain was rated higher during NPs (T2 and T5) both by the patients and by the observers (who had no access to data reported by the patient) as compared to assessments at rest. These data show good discriminant validity of the POL-BPS.

**Table 8:**
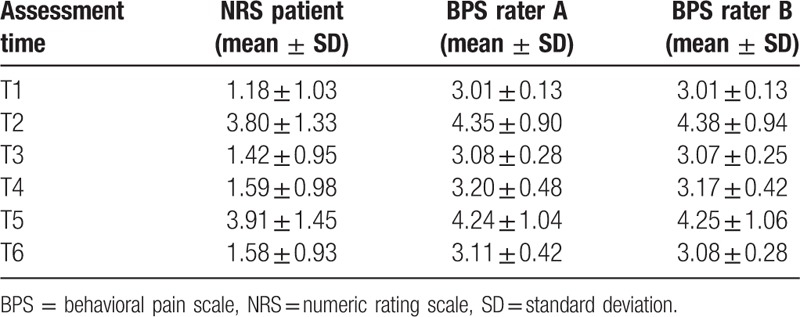
Distribution of BPS and NRS results at predetermined assessment times (T1–T6).

### 3.4 Criterion validity for POL-BPS

The data in Table 3 show relatively good correlation between patient's self-reported pain (NRS at T2 and T5) and pain observed by rater A and rater B with the use of BPS (Rs > 0.57, *P* < .00001). The results of NRS were available for all 59 patients.

**Figure 2:**
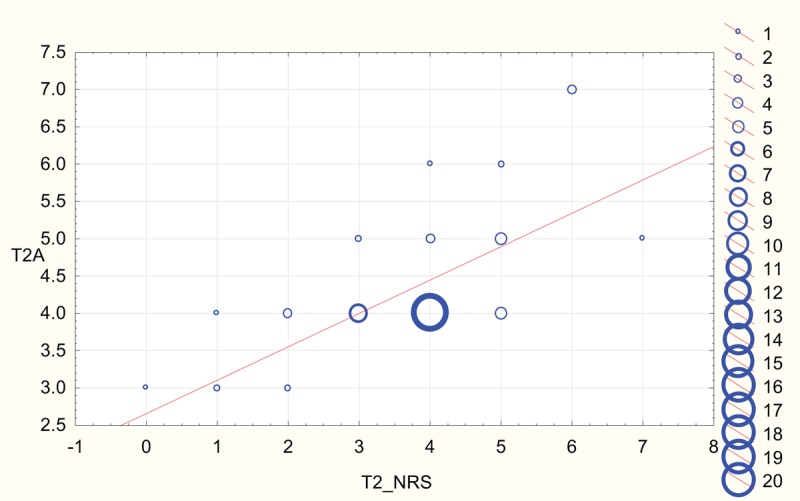
Scatterplot depicting correlation between numeric rating scale (NRS) and behavioral pain scale reported at T2 by rater A (T2A). The diameter of the circles is proportional to the number of values at respective coordinates.

**Figure 3:**
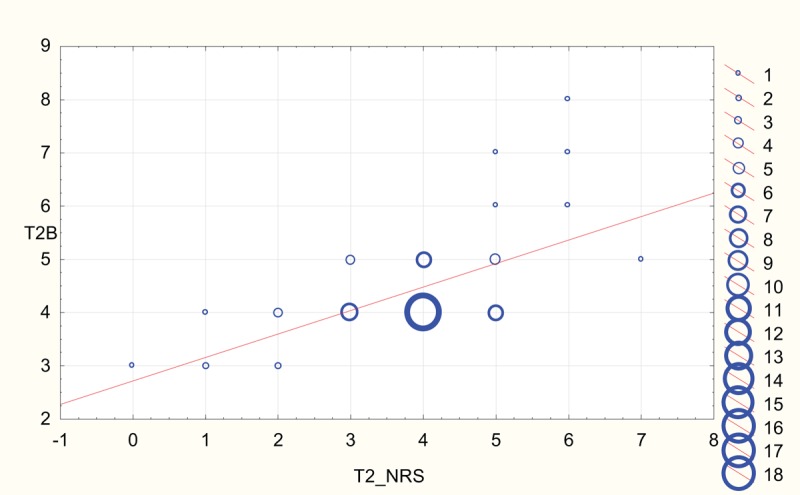
Scatterplot depicting correlation between numeric rating scale (NRS) and behavioral pain scale reported at T2 by rater B (T2B). The diameter of the circles is proportional to the number of values at respective coordinates.

**Figure 4:**
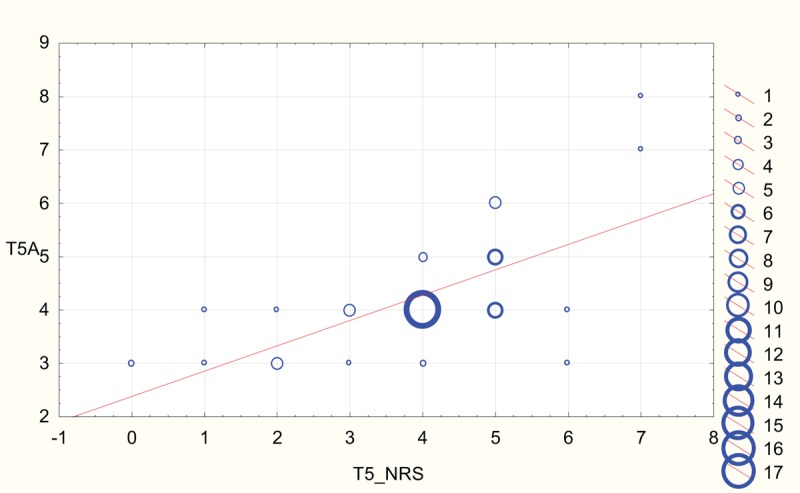
Scatterplot depicting correlation between numeric rating scale (NRS) and behavioral pain scale reported at T5 by rater A (T5A). The diameter of the circles is proportional to the number of values at respective coordinates.

**Figure 5:**
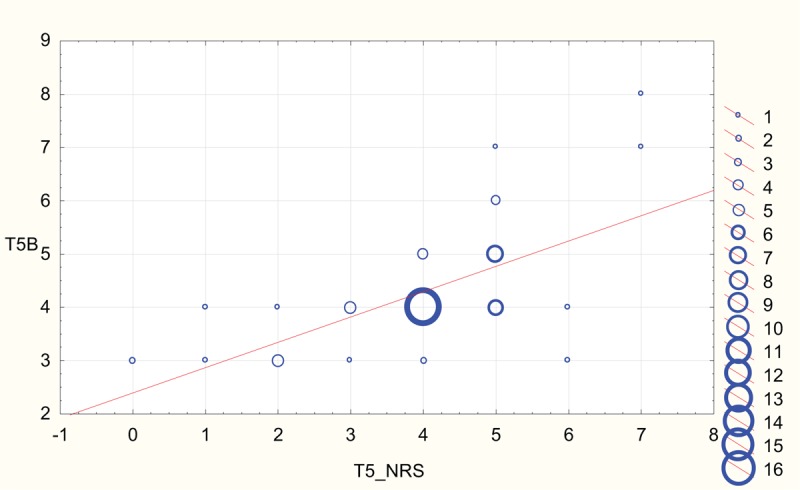
Scatterplot depicting correlation between numeric rating scale (NRS) and behavioral pain scale reported at T5 by rater B (T5B). The diameter of the circles is proportional to the number of values at respective coordinates.

Scatterplots depicting strong correlation between BPS and NRS results for both rater A and rater B at T2 (NP in sedated patients) and T5 (NP in unsedated patients) are shown in Figures 2–5.

### 3.5 Correlation between BPS and physiologic parameters

Spearman rank correlation coefficients for tested physiologic parameters (heart rate, systolic arterial blood pressure, diastolic arterial blood pressure, and respiratory rate) and patients’ NRS score and BPS score for rater A and rater B are shown in Table 4. The analysis shows that significant (*P* < .05) correlations exist only between systolic blood pressure or respiratory rate and NRS during T2 and between respiratory rate and BPS for rater A at T2, but they do not pass Bonferroni correction (*P* > .00096). Overall no statistically significant correlation was found between physiologic parameters and BPS.

## 4 Discussion

The POL-BPS study was performed to validate the POL-BPS in a special population of patients undergoing cardiac surgery who were assessed both sedated and unsedated, but intubated and mechanically ventilated at time of data collection. Two important elements occurred in this study. First, we have performed a validation of the POL-BPS. Second, we were able to reliably assess pain in sedated postoperative cardiac patients using BPS and their self-assessment.

According to international recommendations and guidelines, adequate pain assessment is a prerequisite not only for optimal pain treatment, but also for effective therapy of agitation and delirium associated with critical illness.^[5,7,12]^ BPSs (CPOT and BPS) were originally available only in French or English, but translation into Polish was performed by the authors of this study.^[10]^ A study validating the Polish version of CPOT has already been published and aids Polish-speaking healthcare professionals and patients worldwide.^[13]^ Our present study concentrated on the validation of the POL-BPS along with other recent attempts to validate the BPS in different countries.^[14–17]^

The results of POL-BPS study showed very high intraclass correlation coefficients (ICC > 0.86), indicating very good interrater reliability of the POL-BPS. Moreover, we have shown good discriminant validity, identified by higher BPS results during painful procedures than at the time before or after these procedures, both with and without sedation. These results indicate that the POL-BPS is a reliable tool for pain assessment.

We have also shown a good correlation between patient's self-reported pain using NRS and pain observed by both raters with the use of BPS (Rs > 0.57) which indicates a relatively good criterion validity (*P* < .00001). Other authors have shown similar results. Ahlers et al have shown a strong correlation between nurses’ BPS ratings and conscious sedated patients’ VRS-4 ratings during the painful procedure (Rs. = 0.67, *P* < .001).^[18]^

The original study performed by Payen et al that introduced BPS to clinical practice showed that it can validly and reliably be used in sedated mechanically ventilated patients. It showed good internal consistency for BPS as determined by standardized Cronbach α=0.89 with good agreement percentages (80%) and acceptable interrater reliability (κ = 0.52–1; ICC = 0.80–0.93).^[11]^ However, the patient group and conditions in their study were different from ours, as the authors included only trauma patients.^[11]^ Our study showed lower internal consistency of the POL-BPS, as Cronbach α during painful procedures ranged from 0.53 to 0.55 for rater A and 0.67 to 0.69 during assessment by rater B. A study performed by Rijkenberg et al also showed lower Cronbach α values than the original study, as their results for the BPS were 0.62 as evaluated by nurse 1 and 0.59 reported by nurse 2.^[19]^ This study included cardiac surgery patients into a comparison between BPS and CPOT and not against NRS, as the patients were unable to self-report pain. In contrast to our study, the type of anesthetic technique and analgesia used intraoperatively and postoperatively by Rijkenberg et al differed greatly between the patients. The ICC reported by them for BPS was 0.74 (95% CI 0.68–0.79), as compared to 0.86 from the results of our study.^[19]^

There are not many studies assessing pain in the postcardiac surgery patient group. The uniqueness of our study lies in the fact that we included observations in both sedated and unsedated patients. Despite receiving sedation (dexmedetomidine) and analgesia (morphine), they were able to interact with the bed-side nurses and raters and self-assess pain using the NRS. Although difficult to obtain in the vulnerable postcardiac surgery period, this led to an appropriate validation of a BPS against patient self-report of pain. Similar recent studies performed in conscious patients suggested that concomitant use of both BPS and CPOT during painful interventions or nursing care may improve the evaluation of pain.^[15]^ Other validation studies of BPS in different patient population and languages have shown similar results.^[18,20–22]^

Moreover, our study group was very homogenous due to a standard anesthesia protocol during the procedure (same set of medications for each patient) and in the postoperative period (dexmedetomidine and morphine was used in all patients). Opioid delivery was a part of routine postoperative analgesia and did not differ between patients, which adds to the homogeneity of the study group. Continuous morphine infusion along with nonopioid analgesics provides a well-balanced analgesia, but may influence patient responsiveness. This was possible in our study, but usually when the patient is able to do self-evaluation, they do not require assessment with a behavioral scale. Some authors suggest that to obtain most reliable results both verbal and nonverbal tools should be used,^[18]^ as this leads to better optimization of the pain treatment in postcardiac surgery patients. Possibly the reason that the correlations between NRS and BPS in our study are below 0.8 is because the NRS and BPS assess pain differently and do not measure exactly the same thing. The assessment of pain should be multi-dimensional and take into account both patient's report as the gold standard (VAS or NRS), as well as observer's report (usually a nurse) using a behavioral scale and other issues, namely patient's previous experience or expectations. It has been much debated recently that carers should move beyond pain scores^[23]^ and acknowledge not only pain intensity measurement, but also patient's personal pain experience, pain threshold, verbal pain evaluation, and opinion. It has been highlighted that some healthcare professionals and patients differ in their interpretation of the postoperative NRS scores, therefore we should take into account the patient's preference for pharmacologic treatment.^[24,25]^

Our study is not without limitations. This observational research was performed in a specific group of patients: postoperative cardiac surgery patients, critically ill people who were intubated and mechanically ventilated. This indicated that this validation study does not cover all potential critically ill patients and that further studies may be required to address specific population (trauma, burn, children, etc). We excluded emergency patients to provide homogeneity of the analyzed population, but there is a need to validate the BPS tool also in this group of patients.

## 5 Conclusion

The results of this study show that the POL-BPS can be regarded as a useful and validated tool for pain assessment in adult intubated patients. This instrument can be used in both unsedated and sedated cardiac surgery patients with limited communication abilities. This study should be regarded as one of the many steps along the way to improve pain assessment and control.

## Acknowledgments

The authors of this study are grateful to the patients, physicians, nurses, and physiotherapists at the Pomeranian Medical University in Szczecin, Poland, for their collaboration. They also thank J.F. Payen for agreeing to allow us to translate the BPS into Polish, and Ms Paulina Spólnicka RN from Teaching Hospital No. 2 for her great effort in data collection. The authors thank Ms Heather Hart RN and Ms Joanna Stollings RN from Vanderbilt University for their kind support with BPS back translation and expert consult.

## Author contributions

KK designed the study, collected the data, analyzed the results and wrote the manuscript. MS collected the data and provided critical review of the manuscript. KS designed and performed statistical analysis. MZB, MŻ, MB, and WE made substantial contributions to the conception of the work and critically reviewed the manuscript. All of the authors read and approved the final manuscript.

**Conceptualization:** Katarzyna Kotfis, Marta Strzelbicka, Małgorzata Zegan-Barańska, Krzysztof Safranow, Mirosław Brykczyński, Maciej Żukowski, Eugene Wesley Ely.

**Data curation:** Katarzyna Kotfis, Marta Strzelbicka, Małgorzata Zegan-Barańska, Krzysztof Safranow.

**Formal analysis:** Katarzyna Kotfis, Marta Strzelbicka, Krzysztof Safranow, Mirosław Brykczyński, Maciej Żukowski, Eugene Wesley Ely.

**Funding acquisition:** Maciej Żukowski.

**Investigation:** Katarzyna Kotfis, Małgorzata Zegan-Barańska.

**Methodology:** Katarzyna Kotfis, Marta Strzelbicka, Małgorzata Zegan-Barańska, Krzysztof Safranow, Eugene Wesley Ely.

**Project administration:** Katarzyna Kotfis.

**Resources:** Katarzyna Kotfis.

**Software:** Katarzyna Kotfis, Marta Strzelbicka.

**Supervision:** Małgorzata Zegan-Barańska, Mirosław Brykczyński, Maciej Żukowski, Eugene Wesley Ely.

**Validation:** Katarzyna Kotfis, Krzysztof Safranow.

**Visualization:** Katarzyna Kotfis, Marta Strzelbicka.

**Writing – original draft:** Katarzyna Kotfis, Krzysztof Safranow.

**Writing – review & editing:** Marta Strzelbicka, Małgorzata Zegan-Barańska, Mirosław Brykczyński, Maciej Żukowski, Eugene Wesley Ely.

Katarzyna Kotfis orcid: 0000-0001-8430-1369
